# A Critical Evaluation of Contextual Factors Affecting the Implementation of Pharmacist-Led Colorectal Cancer Screening: A Scoping Narrative Hybrid Review

**DOI:** 10.5888/pcd23.250380

**Published:** 2026-03-26

**Authors:** Arinze Nkemdirim Okere, Md. Mohaimenul Islam

**Affiliations:** 1 Division of Outcomes and Practice Advancement, Department of Pharmacy Practice and Science, School of Pharmaceutical Sciences, University at Buffalo, New York

## Abstract

**Introduction:**

Colorectal cancer (CRC) is among the most preventable cancers and causes more than 50,000 deaths annually in the US. Screening disparities persist in rural versus urban populations and in populations with inadequate access to care. Community pharmacies, given their accessibility and trusted role in preventive care, represent a promising setting to expand CRC screening.

**Methods:**

This scoping narrative hybrid review followed the Preferred Reporting Items for Systematic Reviews and Meta-Analyses Extension for Scoping Reviews (PRISMA-ScR) guidelines and the Arksey and O’Malley framework, along with other refinements. Literature searches were conducted in PubMed, Embase (via Elsevier), ProQuest, Medline (via OVID), and ClinicalTrials.gov by using broad key words related to CRC screening and pharmacy. Eligible studies included US-based studies describing pharmacist involvement in community pharmacy CRC screening. Data were synthesized by using the Consolidated Framework for Implementation Research, with strategies mapped to the Implementation Research Logic Model.

**Results:**

Ten US studies met inclusion criteria: 1 randomized community–pharmacy intervention trial, 1 pilot implementation study, 3 stakeholder qualitative studies, and 5 national or statewide surveys. Key barriers included lack of reimbursement, workflow constraints, pharmacist knowledge gaps, and limited care coordination. Facilitators included high patient willingness, trust in pharmacies, compatibility with existing workflows, and professional support from pharmacists and providers. Evidence-based strategies included targeted pharmacist training, workflow reminders, formal referral agreements with primary care, and patient engagement initiatives. Policy reforms to establish reimbursement and strengthen information exchange were identified as essential for sustainability.

**Conclusion:**

Community pharmacies are well positioned to expand CRC screening access. Addressing reimbursement, training, workflow, and coordination barriers through evidence-based strategies and supportive policy can enable pharmacist-delivered CRC screening to reduce disparities and improve population health outcomes.

**Summary**

**What is known about this topic?**

Colorectal cancer (CRC) is among the most frequently diagnosed cancers in the US, and screening disparities persist in rural populations and in populations with inadequate access to care. Community pharmacies represent a promising setting to expand CRC screening.

**What is added by this report?**

We conducted a literature review to examine the barriers to and facilitators of pharmacy-based CRC screening. Key barriers included lack of reimbursement, workflow constraints, pharmacist knowledge gaps, and limited care coordination. Facilitators included high patient willingness, trust in pharmacies, compatibility with existing workflows, and professional support from pharmacists and providers.

**What are the implications for public health practice?**

Community pharmacies are well positioned to expand CRC screening access to reduce disparities and improve population health.

## Introduction

Colorectal cancer (CRC) is both common and preventable. It is the third most frequently diagnosed cancer worldwide and the fourth in the US, causing more than 50,000 deaths annually (1–4). Although screening can detect CRC early and reduce mortality rates, incidence continues to increase among younger adults, leading the US Preventive Services Task Force to lower the recommended starting age for average-risk adults to 45 years (5,6).

Despite these guidelines, screening rates remain uneven. People in rural and underserved urban areas are less likely to be screened, contributing to later diagnoses and poorer survival outcomes (7,8). Access barriers include shortages of primary care providers (PCPs), long travel distances, and limited oncology services. Social and behavioral factors such as lower income, lower education, unemployment, smoking, and physical inactivity are associated with less screening use (9–12). Together, these challenges highlight the need for new approaches to expand access and improve equity.

Less invasive tests, including fecal immunochemical tests (FITs) and stool DNA tests (eg, Cologuard), are accurate, convenient, and easier to complete than colonoscopy (6). However, use of these tests among underserved groups remains disproportionately low (7,8). This gap shows that making tests available is not enough; they must also be accessible where patients seek care, and patients must be provided education and clear instructions on how to ensure their correct use and timely return. 

Community pharmacies offer a promising solution. More than 90% of people in the US live within 5 miles of a pharmacy, making them highly accessible (13,14). In a national survey, 72% of adults aged 45 to 75 years expressed willingness to obtain a FIT from a pharmacy (15). Additionally, the American Society of Health-System Pharmacists recognizes that community pharmacists “can provide direct patient care, advance team-based care, manage patient-centered clinical services, and serve as leaders within their communities and health systems” (16). These capabilities have been demonstrated across multiple studies showing that community pharmacist engagement significantly improves use of preventive services, most notably adult vaccinations (17,18). Together, these strengths position community pharmacies as strategic partners for expanding CRC screening.

Therefore, the objective of this review was to critically examine the barriers to and facilitators of pharmacy-based CRC screening and identify strategies to support effective implementation.

## Methods

This scoping narrative hybrid review followed the Preferred Reporting Items for Systematic Reviews and Meta-Analyses (PRISMA) Extension for Scoping Reviews (PRISMA-ScR) guidelines to provide an overview of contextual factors influencing the implementation of CRC screening in community pharmacies. The review approach was guided by the scoping framework developed by Arksey and O’Malley (19) and further advanced by Levac et al (20).

We addressed 2 primary questions:

1. What barriers and facilitators influence the implementation of CRC screening in community pharmacies?
2. What evidence-based strategies can address these barriers and enhance facilitators?

### Literature search and study selection

A comprehensive literature search was performed in PubMed, Embase (via Elsevier), and Medline (via OVID) through October 2025. Search strategies were developed iteratively and included both controlled vocabulary (MeSH/Emtree) and free-text key words. Core terms used were *“colorectal cancer screening” OR “CRC screening” and Pharmacist; “colorectal cancer screening” OR “CRC screening” and Pharmacy.* In ProQuest, we used the search terms *“Pharmacy and (Colorectal Cancer Screening) and Barriers and Facilitators.”* Finally, we conducted supplementary searches in ClinicalTrials.gov to identify registered, completed, and unpublished trials. No language or date restrictions were applied to maximize retrieval of relevant evidence.

After removing duplicates, we independently screened titles and abstracts, followed by full-text review. Disagreements were resolved through discussion. Only studies published in English were considered.

### Inclusion and exclusion criteria

Eligible studies included randomized and nonrandomized controlled trials, observational cohorts, case–control studies, cross-sectional surveys, and descriptive studies. Editorials and case reports were excluded.

We included studies conducted in the US and evaluating pharmacist provision of CRC screening in community pharmacy settings. Studies in nonpharmacy settings were excluded.

The primary outcome was identification of barriers to and facilitators of CRC screening implementation in community pharmacies, mapped to the Consolidated Framework for Implementation Research (CFIR) domains. The secondary outcome was the identification of potential evidence-based strategies, guided by the Expert Recommendations for Implementing Change framework (21).

### Data extraction and synthesis

Data were extracted by using a predefined form summarizing study characteristics, outcomes, and implementation factors. Consistent with scoping review methods, we did not appraise the quality of included studies. Extracted data were organized by using CFIR to categorize barriers and facilitators. Because some studies did not explicitly report CFIR domains, we deductively coded relevant findings to CFIR constructs. We organized implementation determinants within CFIR and mapped them to corresponding strategies by using the Implementation Research Logic Model (IRLM) (22).

### Role of narrative synthesis

To minimize subjectivity during synthesis, all data extraction and CFIR coding followed a structured and deductive process. We extracted implementation determinants from each included study and mapped them to CFIR domains by using predefined coding rules, ensuring consistent application across all 10 studies. In addition to mapping findings through CFIR and IRLM, we integrated a narrative synthesis to interpret results across diverse study designs and populations. This approach allowed us to compare how barriers and facilitators manifested across settings, highlight recurring patterns, and situate US pharmacy-based studies within a broader international context. The narrative component strengthened our ability to draw practice-oriented insights and implementation strategies that extend beyond descriptive mapping.

## Results

### Study selection and scope

**Figure 1:**
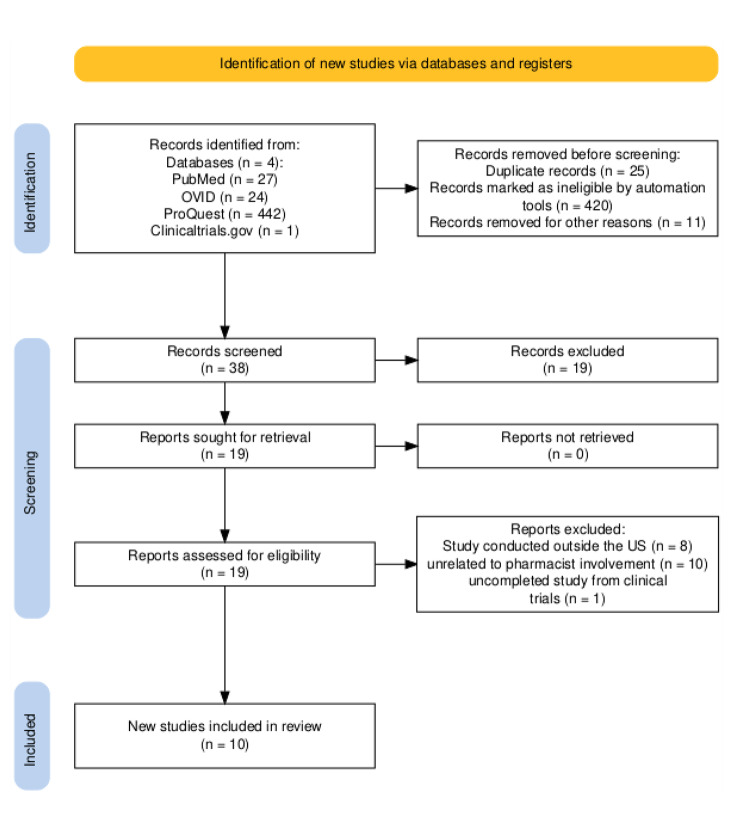
Flowchart showing the identification of new studies via databases and registers through 2025 in a scoping narrative hybrid review of factors affecting pharmacist-led colorectal cancer screening. Source: Haddaway et al (32).

A total of 494 records were identified across databases and trial registries. After removal of duplicates, automated exclusions, and other ineligible records, 38 records were screened. Nineteen full-text reports were reviewed, of which 18 were excluded (Figure 1). Ten studies met eligibility criteria and were included in the final review (Figure 1). Of these, 9 US studies directly examined community pharmacy–based CRC screening: 1 time-randomized community-pharmacy intervention trial, 1 pilot feasibility study, 3 stakeholder qualitative studies (patients, pharmacists, and PCPs), and 4 national surveys addressing patient willingness, pharmacist perceptions, pharmacist knowledge, and patient-reported barriers (15,23–30). One additional US population-level analysis of CRC screening determinants, although not community pharmacy–specific, informed contextual interpretation of patient-level factors such as age, insurance, and socioeconomic gradients (31). An overview of study characteristics and findings is provided in the Table.

**Table:** Selected Studies on the Contextual Factors Impacting CRC Screening in Community Pharmacies

Author, year	Country/setting	Age, y	Design/sample	Objective	Outcomes	Key findings	CFIR domains
Potter et al, 2010 (23)	US/community pharmacies during influenza vaccination	50–80	Time-randomized trial; n = 133 eligible adults	Compare education-only vs on-site FIT kit distribution during pharmacy influenza clinics	FIT completion; reasons for noncompletion; acceptability	FIT completion 59.3% (FIT arm) vs 14.8% (education only), *P* < .001; top noncompletion reasons included forgetting (~29%) and losing kits (~20%).	Intervention characteristics; outer setting; process
Holle et al, 2020 (24)	US/Connecticut independent-chain pharmacies	NR (adult participants)	Prospective pilot; 312 approached, 16 consented; 8 opted for FIT; 88% of FITs returned correctly	Assess feasibility of a pharmacist-led CRC counseling and FIT service for underserved patients	Recruitment/uptake; FIT return; satisfaction; workflow feasibility	Very small uptake; high FIT return among those who accepted; pharmacist training/time and lack of reimbursement were major implementation barriers.	Intervention characteristics; inner setting; process
Brenner et al, 2023 (25)	US/primary care (North Carolina and Washington)	Not applicable (PCPs)	Qualitative interviews; n = 30 PCPs	Elicit PCP views on PharmFIT and requirements for safe implementation	Themes: interoperability, follow-up ownership, training	Broad support if closed-loop communication, clear responsibility for positive FIT follow-up, and EHR interoperability are ensured.	Outer setting; inner setting; process
Ferrari et al, 2023 (26)	US/patients (North Carolina and Washington)	Screening-eligible adults (NR)	Qualitative interviews; n = 32 adults	Understand patient acceptability and design preferences for pharmacy-based FIT	Acceptability; perceived barriers; privacy/trust	Patients found PharmFIT acceptable for access/convenience; emphasized privacy, coordination with PCPs, and closed-loop care.	Intervention characteristics; outer setting; inner setting
Waters et al, 2024 (27)	US/community pharmacists	Not applicable (pharmacists)	Qualitative study; n = ~25 community pharmacists	Identify pharmacist recommendations to design/implement PharmFIT	Feasibility; resource needs; reimbursement	Feasible with SOPs, training, and clear reimbursement; stressed EHR connectivity, and defined roles for positive FIT follow-up.	Intervention characteristics; outer setting; inner setting
Shah et al, 2024 (15)	US/national	45–75	National online survey; n = 1,045 adults	Measure willingness to use PharmFIT and correlates (DOI traits)	Willingness (primary); predictors	72% Willing to use PharmFIT; willingness increased with perceived relative advantage (β ≈ 0.184) and compatibility (β ≈ 0.422); lower among those aged ≥65 y.	Intervention characteristics; outer setting; inner setting; characteristics of individuals
Urbanek et al, 2024 (28)	US/Kentucky community pharmacists	Not applicable (pharmacists)	Statewide survey; n = 207 responses (151 community-based)	Assess support and barriers to offering CRC screening via board-authorized protocol	Support rate; training needs; barrier prevalence	34% Agreed/strongly agreed to offer screening; 81.3% need more training; top barriers: lack of reimbursement (97%), patients’ unwillingness to pay (98%), time/workflow (97%).	Intervention characteristics; inner setting; process
Odebunmi et al, 2025 (29)	US/national community pharmacists	Not applicable (pharmacists)	National survey; n ≈ 578 pharmacists	Assess pharmacists’ CRC screening knowledge and training needs	Knowledge of age/start interval; knowledge gaps	Many knew basic concepts (eg, colonoscopy after positive FIT, FIT is home-based) but large gaps for start age (45 y) and interval; board certification associated with higher knowledge.	Characteristics of individuals; inner setting
Bromm et al, 2023 (31) (contextual)	US/Michigan population (MiBRFSS 2018)	≥50	Cross-sectional state survey	Correlation between age and rurality on CRC screening to inform pharmacist focus areas	Self-reported stool tests and colonoscopy; predictors	Older age groups had higher odds of being up-to-date; lack of insurance decreased screening; higher education/income increased screening.	Outer setting
Schwartz et al, 2025 (30)	US/National	45–75	National survey; n = 1,045 adults	Identify preferences for follow-up care and barriers after positive FIT in a pharmacy-based program	Preferred communication modes; psychosocial vs structural barriers	Digital communications preferred for negative results/reminders; direct provider contact preferred for positive results; psychosocial barriers (eg, fear, anxiety) more common than structural; structural barriers lower among those with a regular provider or recent CRC screening.	Intervention characteristics; outer setting; characteristics of individuals; process

Abbreviations: CFIR, Consolidated Framework for Implementation Research; CRC, colorectal cancer; DOI, digital object identifier; EHR, electronic health record; FIT, fecal immunochemical test; MiBRFSS, Michigan Behavioral Risk Factor Surveillance System; PCPs, primary care providers; SOPs, standard operating procedures.

### CFIR-organized synthesis

#### Intervention characteristics

In a time-randomized trial during influenza vaccination clinics, direct provision of FITs in pharmacies produced significantly higher completion than education alone (59.3% vs 14.8%, *P* < .001). Noncompletion was mostly behavioral — forgetting (29%) or losing (20%) kits — while only 5.7% cited complicated instructions, showing low intrinsic complexity (Potter et al, 2010) (23). A pilot feasibility study confirmed that when patients accepted PharmFIT, 88% completed the test correctly, reinforcing usability and patient satisfaction (Holle et al, 2020) (24). National survey data further showed patients perceive PharmFIT as convenient, compatible with routine care, and low in complexity, reinforcing its relative advantage over clinic-based pathways (Shah et al, 2024) (15). Interviews and pharmacist feedback echoed this view, emphasizing that clear instructions and simple packaging could minimize barriers (26,27).

Stakeholders consistently highlighted user-friendly design as critical. Patients and pharmacists preferred 1-page instructions or short videos, discreet packaging, and easy return processes. These features were linked to higher acceptability and likelihood of FIT completion (23,24,26,27).

Cost concerns were common across audiences. In the trial, only 38.6% of participants were willing to pay $20 or more for a FIT kit (23). Pharmacists identified lack of reimbursement (97%) and patients’ unwillingness to pay (98%) as dominant barriers, with fewer than half believing PharmFIT could be sustained economically under current conditions (28).

#### Outer setting

Approximately 72% of adults nationally were willing to participate in PharmFIT, with willingness exceeding 95% when programs guaranteed physician endorsement, routed results to primary care, and had no or low-cost coverage (15). Patient interviews described pharmacies as trusted, accessible venues that reduce travel and appointment constraints, especially for patients with limited access to care (26). A national survey found that patients preferred digital communication for negative results but direct provider contact for positive results, with 93% preferring physicians to make colonoscopy referrals (30). At the population level, older US adults were more likely to have ever been screened but were less likely to be up to date, and uninsured patients had much lower screening rates. Higher education and income were associated with increased use (31).

PCPs supported PharmFIT as complementary to clinic-based screening but emphasized the need for closed-loop communication and clear responsibility for positive FIT follow-up. Limited electronic health record interoperability and reliance on fax machines or telephones for communication were seen as safety risks (25). Pharmacists reported uncertainty about ordering authority, billing, and payer rules, particularly when pharmacies were out of network (27).

#### Inner setting

Pharmacies showed structural compatibility for CRC screening by leveraging existing immunization and point-of-care workflows (23,27). However, implementation remained rare. In a national survey, 88% of pharmacists reported access to prescriber-signed protocols, but only 0.04% had actually implemented PharmFIT (28). Workflow burden (97%) and lack of reimbursement were the main barriers.

Pharmacists consistently requested training and standardized processes or standard operating procedures (SOPs). In a national survey, only 34% correctly identified the updated start age (45 years) and 28% the annual FIT interval; only 5% answered all items correctly. Knowledge was higher among board-certified pharmacists (29). Both pharmacists and PCPs emphasized the need for clear workflows and interoperable systems to ensure safe follow-up (25,27).

#### Characteristics of individuals

Patients trusted pharmacists for eligibility and education but wanted PCPs involved in positive results and colonoscopy referrals. National survey data showed 76% of adults were comfortable with pharmacist counseling, 71% with reminders, and 67% with eligibility discussions, but only 53% supported pharmacists discussing positive results and 39% referrals (Shah et al, 2024) (15). Interviews confirmed this, with patients emphasizing the “gold standard” role of colonoscopy and physician involvement (26,30).

Pharmacists’ knowledge varied widely: most knew FIT was home-based (84%) and that colonoscopy followed a positive result (87%), but fewer recognized the start age (34%) or interval (28%) and only 5% answered all questions correctly. Knowledge improved with years in practice and board certification (29).

Pharmacists expressed moderate confidence in using protocols and counseling but high demand for training (81%). Willingness to implement did not vary by age, sex, years in practice, or prescription volume (28). In the trial, behavioral barriers like forgetting or misplacing kits drove noncompletion, showing the importance of reminders (23).

#### Process

Pharmacies effectively engaged patients during routine encounters. In the trial, 10% to 12% of adults in vaccination lines consented to PharmFIT, demonstrating feasibility (23). Patients were open to pharmacist-initiated conversations when privacy and discretion were ensured (26). National willingness was associated with perceptions of compatibility and relative advantage (15).

Pharmacy-embedded distribution produced higher completion than education-only approaches (59.3% vs 14.8%, *P* < .001) (23). Yet pharmacists and PCPs stressed the need for SOPs, clear referral pathways, and interoperable systems to prevent loss to follow-up (25,27).

Safe implementation requires defined communication roles. PCPs supported PharmFIT but insisted on closed-loop systems and physician responsibility for positive results (25). Pharmacists echoed concerns about unclear routing and billing (27). Patients preferred texts or emails for negative results but direct provider contact for positive results, with nearly all expecting physicians — not pharmacists — to arrange colonoscopy (30).

Stakeholders recommended monitoring kit distribution, return rates, time to colonoscopy after a positive FIT, and workflow efficiency as part of continuous improvement (27,28).

**Figure 2:**
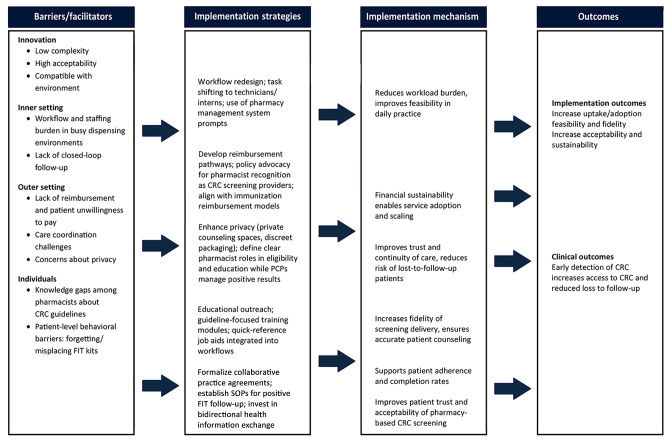
Contextual factors affecting colorectal cancer (CRC) screening in community pharmacy settings and implementation strategies identified in a scoping narrative hybrid review of factors affecting pharmacist-led CRC screening. Abbreviations: FIT, fecal immunochemical test; PCPs, primary care providers; SOPs, standard operating procedures.

To demonstrate how barriers, facilitators, and strategies align across levels of implementation, findings were organized within CFIR domains and mapped to IRLM (Figure 2).

## Discussion

This scoping narrative hybrid review examined barriers to, facilitators of, and strategies for implementing CRC screening in US community pharmacies. Findings suggest that pharmacies have strong potential to expand access, but sustained implementation will require policy change, workflow adaptations, and stronger collaboration with primary care.

### Barriers to implementation

The most pressing barrier was financial. Nearly all pharmacists reported that absence of payment mechanisms, coupled with patients’ unwillingness to pay out of pocket, made the service financially unsustainable (28). This finding was reinforced by evidence from Potter et al, which found that only approximately one third of patients were willing to pay more than $20 for a FIT kit (23). Additionally, stool DNA tests (eg, Cologuard) are becoming increasingly available, with a reported list price of approximately $600 per test. Although many insured patients pay little or nothing out of pocket, people who are uninsured or underinsured may be responsible for the full cost. This combination of high nominal pricing and inconsistent coverage raises concerns about equity and feasibility. Therefore, without reimbursement pathways, pharmacists cannot sustain CRC screening services.

Workflow demands were another challenge; pharmacists described CRC screening as time consuming and difficult to integrate into busy dispensing environments (28). Knowledge gaps also limit fidelity, as many pharmacists are unaware of key screening guidelines (29). Finally, limited communication with primary care creates uncertainty about who should manage positive results, raising care coordination concerns (25,26)

An additional concern relates to test performance. In a prospective observational study conducted by Imperiale et al (2024) involving 20,176 participants, the next-generation multitarget stool DNA test demonstrated a sensitivity of 93.9% (95% CI, 87.1%–97.7%) for CRC and 43.4% (95% CI, 41.3%–45.6%) for advanced precancerous lesions, with a specificity of 90.6% (95% CI, 90.1%–91.0%) for advanced neoplasia. In contrast, FIT showed lower sensitivity of 67.3% CRC and 23.3% for advanced precancerous lesions (33). Thus, with the positive predictive value being modest and only a minority of positive results indicating advanced neoplasia, many people will require follow-up colonoscopy and subsequent care that extends beyond the scope of community pharmacy practice. These realities underscore the need for robust pretest counseling, clear communication of results, and structured referral pathways — elements that may exceed the capacity of pharmacists working without collaborative agreements. Simply advising patients to “see your primary care doctor” is insufficient without a defined referral mechanism and timely bidirectional communication system.

### Facilitators of implementation

Despite these challenges, several factors support feasibility. Patients consistently view pharmacies as accessible and trustworthy, and most express willingness to complete a stool-based test if available in this setting (15,26). Pharmacies already deliver immunizations and point-of-care testing, providing an operational model that can be extended to CRC screening (23,27). Both pharmacists and PCPs recognize the public health value of pharmacy-based CRC screening. Specifically, PCPs viewed pharmacy-based CRC screening as complementary to clinic-based testing and a way to reach unscreened patients (25).

### Strategies and policy implications

Consistent with the principle of using the right screening test for the right population at the right interval, current US Preventive Services Task Force recommendations call for CRC screening in average-risk adults aged 45 to 75 years, with stool-based options including annual FITs or high-sensitivity guaiac testing, and stool DNA–FIT every 1 to 3 years. Because the prevalence of CRC in asymptomatic, average-risk adults is low, even highly sensitive stool DNA tests will demonstrate only modest positive predictive value. This highlights the importance of offering stool DNA–FIT to guideline-concordant populations — adults aged ≥45 years who are due for screening — and using alternative strategies when clinically indicated. The cost of stool DNA tests and the possibility of false positives also raise practical and ethical considerations that reinforce the need for thoughtful implementation, clear patient communication, and structured follow-up processes. In this context, standardized eligibility screening and stronger linkage between pharmacy-based screening and primary care or public health programs may help support safe and appropriate use.

Specific training and decision aids may help pharmacists provide guideline-aligned counseling, while workflow prompts and reminders could improve test completion rates. Implementation of stool DNA–FIT will also benefit from clear protocols addressing patient eligibility, communication of positive results, and referral pathways. Collaborative arrangements with PCPs and, when appropriate, local public health departments can help ensure timely follow-up and shared responsibility for patient care. Given the cost and moderate predictive value of stool DNA–FIT, engagement with payers and public health partners may be important to support responsible and equitable implementation.

At the policy level, reimbursement reform is essential to make pharmacy-based CRC screening financially sustainable. Investments in health information technology can further support coordination and ensure closed-loop care between pharmacies and primary care. Embedding pharmacy-based CRC screening into national initiatives, such as the Centers for Disease Control and Prevention’s Colorectal Cancer Control Program, could accelerate adoption and promote equity in underserved populations.

Although several considerations must be addressed, the key message is that community pharmacies remain a promising and accessible setting for expanding CRC screening. Strengthening bidirectional communication and coordination between community pharmacies and PCPs can help navigate clinical complexities, ensure appropriate follow-up, and ultimately increase screening uptake in ways that complement and reinforce existing care pathways.

### Strengths, limitations, and future directions

This review is the first to synthesize US evidence on pharmacy-based CRC screening using CFIR, capturing perspectives of patients, pharmacists, and providers. However, as a scoping narrative review, it did not assess study quality, and evidence remains limited to small trials, surveys, and pilot studies. Future work should prioritize large-scale demonstration projects, policy evaluation, and outreach for underserved groups, especially rural, low-income, and uninsured populations.

### Conclusion

Community pharmacies are well positioned to expand CRC screening access. High patient willingness, professional support, and compatibility with existing services provide a strong foundation. Addressing reimbursement, workflow, and care coordination challenges will be critical to realizing the full potential of pharmacy-based CRC screening as a tool to reduce disparities and improve population health.

## References

[1] Morgan E , Arnold M , Gini A , Lorenzoni V , Cabasag CJ , Laversanne M , . Global burden of colorectal cancer in 2020 and 2040: incidence and mortality estimates from GLOBOCAN. *Gut.* 2023;72(2):338–344. 10.1136/gutjnl-2022-327736 36604116

[2] National Cancer Institute. Cancer stat facts: colorectal cancer. Surveillance, Epidemiology and End Results Program. Accessed July 23, 2025. https://seer.cancer.gov/statfacts/html/colorect.html

[3] Keum N , Giovannucci E . Global burden of colorectal cancer: emerging trends, risk factors and prevention strategies. *Nat Rev Gastroenterol Hepatol.* 2019;16(12):713–732. 10.1038/s41575-019-0189-8 31455888

[4] Sokale IO , Rosales O , Montealegre JR , Oluyomi AO , Thrift AP . Trends in up-to-date colorectal cancer screening among US adults aged 50–75 years and variations by race/ethnicity and US Census Bureau divisions. *AJPM Focus.* 2022;2(1):100055. 10.1016/j.focus.2022.100055 37789945 PMC10546535

[5] Sifaki-Pistolla D , Poimenaki V , Fotopoulou I , Saloustros E , Mavroudis D , Vamvakas L , . Significant rise of colorectal cancer incidence in younger adults and strong determinants: 30 years longitudinal differences between under and over 50s. *Cancers (Basel).* 2022;14(19):4799. 10.3390/cancers14194799 36230718 PMC9563745

[6] Davidson KW , Barry MJ , Mangione CM , Cabana M , Caughey AB , Davis EM , ; US Preventive Services Task Force. Screening for colorectal cancer: US Preventive Services Task Force recommendation statement. *JAMA.* 2021;325(19):1965–1977. 10.1001/jama.2021.6238 34003218

[7] Anderson AE , Henry KA , Samadder NJ , Merrill RM , Kinney AY . Rural vs urban residence affects risk-appropriate colorectal cancer screening. *Clin Gastroenterol Hepatol.* 2013;11(5):526–533. 10.1016/j.cgh.2012.11.025 23220166 PMC3615111

[8] Primm KM , Malabay AJ , Curry T , Chang S . Who, where, when: colorectal cancer disparities by race and ethnicity, subsite, and stage. *Cancer Med.* 2023;12(13):14767–14780. 10.1002/cam4.6105 37212502 PMC10358189

[9] Chen WH , Bloom RD , Brandford A , Han G , Horel S , Sanchez M , . Persistent poverty, rural location, and racial segregation are factors in colorectal cancer screening in low-income and uninsured populations. *J Adv Pract Oncol.* 2025;16(7):1–13. 40599196 10.6004/jadpro.2025.16.7.14 PMC12207529

[10] Sepassi A , Li M , Zell JA , Chan A , Saunders IM , Mukamel DB . Rural–urban disparities in colorectal cancer screening, diagnosis, treatment, and survivorship care: a systematic review and meta-analysis. *Oncologist.* 2024;29(4):e431–e446. 10.1093/oncolo/oyad347 38243853 PMC10994268

[11] Wilkins T , Gillies RA , Harbuck S , Garren J , Looney SW , Schade RR . Racial disparities and barriers to colorectal cancer screening in rural areas. *J Am Board Fam Med.* 2012;25(3):308–317. 10.3122/jabfm.2012.03.100307 22570394

[12] Hughes AG , Watanabe-Galloway S , Schnell P , Soliman AS . Rural–urban differences in colorectal cancer screening barriers in Nebraska. *J Community Health.* 2015;40(6):1065–1074. 10.1007/s10900-015-0032-2 25910484 PMC4620062

[13] Berenbrok LA , Tang S , Gabriel N , Guo J , Sharareh N , Patel N , . Access to community pharmacies: a nationwide geographic information systems cross-sectional analysis. *J Am Pharm Assoc (Wash DC).* 2022;62(6):1816–1822.e2. 10.1016/j.japh.2022.07.003 35965233

[14] Qato DM , Zenk S , Wilder J , Harrington R , Gaskin D , Alexander GC . The availability of pharmacies in the United States: 2007–2015. *PLoS One.* 2017;12(8):e0183172. 10.1371/journal.pone.0183172 28813473 PMC5559230

[15] Shah PD , Wangen M , Rohweder CL , Waters AR , Odebunmi OO , Marciniak MW , . Patient willingness to use a pharmacy-based colorectal cancer screening service: a national survey of US adults. *Cancer Epidemiol Biomarkers Prev.* 2024;33(1):63–71. 10.1158/1055-9965.EPI-23-0763 37909917 PMC10842686

[16] Ortega M , Isom C , Place A , Rush J , Storvick Boedecker A , Luchen GG , . ASHP statement on the community pharmacist’s role in the care continuum. *Am J Health Syst Pharm.* 2025;82(21):e923–e929. 10.1093/ajhp/zxaf146 40627500

[17] Ban A , Shrestha A , Van-den Berk Clark C , Ballard J , Logan R Jr , Logan T Sr , . Stronger together: a community case study highlighting the benefits of pharmacy and community collaborations. *JAPhA Pract Innov.* 2025;2(1):100024. 10.1016/j.japhpi.2024.100024

[18] Le LM , Veettil SK , Donaldson D , Kategeaw W , Hutubessy R , Lambach P , . The impact of pharmacist involvement on immunization uptake and other outcomes: an updated systematic review and meta-analysis. *J Am Pharm Assoc (Wash DC).* 2022;62(5):1499–1513.e16. 10.1016/j.japh.2022.06.008 35961937 PMC9448680

[19] Arksey H , O’Malley L . Scoping studies: towards a methodological framework. *Int J Soc Res Methodol.* 2005;8(1):19–32. 10.1080/1364557032000119616

[20] Levac D , Colquhoun H , O’Brien KK . Scoping studies: advancing the methodology. *Implement Sci.* 2010;5(1):69. 10.1186/1748-5908-5-69 20854677 PMC2954944

[21] Powell BJ , Waltz TJ , Chinman MJ , Damschroder LJ , Smith JL , Matthieu MM , . A refined compilation of implementation strategies: results from the Expert Recommendations for Implementing Change (ERIC) project. *Implement Sci.* 2015;10(1):21. 10.1186/s13012-015-0209-1 25889199 PMC4328074

[22] Smith JD , Li DH , Rafferty MR . The Implementation Research Logic Model: a method for planning, executing, reporting, and synthesizing implementation projects. *Implement Sci.* 2020;15(1):84. 10.1186/s13012-020-01041-8 32988389 PMC7523057

[23] Potter MB , Gildengorin G , Wang Y , Wu M , Kroon L . Comparative effectiveness of two pharmacy-based colorectal cancer screening interventions during an annual influenza vaccination campaign. *J Am Pharm Assoc (Wash DC).* 2010;50(2):181–187. 10.1331/JAPhA.2010.09199 20199960

[24] Holle LM , Levine J , Buckley T , White CM , White C , Hadfield MJ . Pharmacist intervention in colorectal cancer screening initiative. *J Am Pharm Assoc (Wash DC).* 2020;60(4):e109–e116. 10.1016/j.japh.2020.02.014 32197754

[25] Brenner AT , Rohweder CL , Wangen M , Atkins DL , Ceballos RM , Correa S , . Primary care provider perspectives on the role of community pharmacy in colorectal cancer screening: a qualitative study. *BMC Health Serv Res.* 2023;23(1):892. 10.1186/s12913-023-09828-3 37612656 PMC10463525

[26] Ferrari RM , Atkins DL , Wangen M , Rohweder CL , Waters AR , Correa S , . Patient perspectives on a proposed pharmacy-based colorectal cancer screening program. *Transl Behav Med.* 2023;13(12):909–918. 10.1093/tbm/ibad057 37756664 PMC10724111

[27] Waters AR , Meehan K , Atkins DL , Ittes AH , Ferrari RM , Rohweder CL , . How pharmacists would design and implement a community pharmacy-based colorectal cancer screening program. *Prev Oncol Epidemiol.* 2024;2(1):2332264. 10.1080/28322134.2024.2332264 38881823 PMC11177275

[28] Urbanek M , Hanna C , Eckmann L , Carr M , Schadler A , Kebodeaux C . Pharmacists perceptions of providing colorectal cancer screening in community-based practice. *J Am Pharm Assoc (Wash DC).* 2024;64(4S):102111. 10.1016/j.japh.2024.102111 38723852

[29] Odebunmi OO , Wangen M , Waters AR , Ferrari RM , Marciniak MW , Rohweder C , . Colorectal cancer screening knowledge among community pharmacists: a national survey. *J Am Pharm Assoc (Wash DC).* 2025;65(1):102130. 10.1016/j.japh.2024.102130 38796158 PMC11584338

[30] Schwartz JL , . Preferences for follow-up care and barriers after positive FIT in a pharmacy-based colorectal cancer screening program. *J Public Health Res.* 2025;44(4):366–379.

[31] Bromm K , Coe AB , Vordenberg SE . The impact of rurality and age on colorectal cancer screening among Michigan residents. *Innov Pharm.* 2023;14(1):10.24926/iip.v14i1.5212. 10.24926/iip.v14i1.5212 38035320 PMC10686677

[32] Haddaway NR , Page MJ , Pritchard CC , McGuinness LA . *PRISMA2020*: an R package and Shiny app for producing PRISMA 2020–compliant flow diagrams, with interactivity for optimised digital transparency and Open Synthesis. *Campbell Syst Rev.* 2022;18(2):e1230. 10.1002/cl2.1230 36911350 PMC8958186

[33] Imperiale TF , Porter K , Zella J , Gagrat ZD , Olson MC , Statz S , ; BLUE-C Study Investigators. Next-generation multitarget stool DNA test for colorectal cancer screening. *N Engl J Med.* 2024;390(11):984–993. 10.1056/NEJMoa2310336 38477986

